# Healthy Advertising Coming to Its Senses: The Effectiveness of Sensory Appeals in Healthy Food Advertising

**DOI:** 10.3390/foods9010051

**Published:** 2020-01-05

**Authors:** Gudrun Roose, Lana Mulier

**Affiliations:** Department of Marketing, Innovation and Organisation, Faculty of Economics and Business Administration, Ghent University, 9000 Ghent, Belgium; Lana.Mulier@UGent.be

**Keywords:** healthy food, sensory marketing, senses, taste perception, thought listing, mental imagery, advertising effectiveness

## Abstract

With increasing obesity rates and the daily overload of unhealthy food appeals, an important objective for advertising today is to promote healthy food consumption. According to previous research, sensory food advertisements referring to multiple senses—a combination of visual (sight), tactile (touch) and olfactory (smell) cues—evoke more positive sensory thoughts and, therefore, higher taste perceptions than advertisements referring to a single sense (e.g., only taste cues). However, this research only focused on sensory advertising for unhealthy food. The current research investigates how sensory advertising can promote healthy food. While multiple-sense ads for unhealthy food were shown to be more effective than single-sense ads, we find that, for healthy food, single-sense ads increase taste perceptions and advertising effectiveness compared to multiple-sense ads. In two laboratory experiments, we show a different underlying process for this effect—that is, single-sense ads evoke fewer negative thoughts than multiple-sense ads, which mediates the effect of single-sense versus multiple-sense ads on taste perceptions and advertising effectiveness. Moreover, we show that these effects occur not only for verbal ads but, importantly, also for visual ads, which are omnipresent today. This article closes with implications for theory and suggestions for food marketers, ad executives, and public policy.

## 1. Introduction

Obesity among adults and children is still an increasing epidemic. The worldwide prevalence of obesity nearly tripled between 1975 and 2016 [1]. However, we know that obesity is preventable by decreasing unhealthy consumption and simultaneously increasing healthy consumption [1]. The extent to which different food products are perceived and classified as healthy versus unhealthy varies greatly (e.g., [2,3]), and there is no generally agreed-upon classification. We define an unhealthy food product as a vice, i.e., a tempting food product that does not imply long-term benefits on consumption. On the other hand, a healthy food product can be defined as a virtue product, which is a product that is not very tempting immediately but is advantageous in the long run [4]. This goal conflict between short-term indulgence and long-term health considerations is at the heart of unhealthy food choices and contributes strongly to overweight [5]. Previous research has often focused on investigating ways to influence people to eat less unhealthy food, such as with information provision, nutrition labels, smaller portions, plates or tables, unit size, package sizes, segmentation cues, etc. [6,7,8,9]. 

To prevent obesity, however, there is not only a need for eating less unhealthy food, but even more importantly, there is a vital need to increase the consumption of healthy food among consumers, especially those at risk of obesity [10,11]. It is, therefore, crucial to upgrade the image of healthy food in the minds of consumers to further attempt to counter the obesity epidemic. Research on the motivational processes underlying the conflict between healthiness and taste is still scarce [5]. A common assumption in existing food research is that consumers want to make healthy food choices, but, in reality, many consumers rather consider taste than the prospective health benefits of the food [12]. As such, a potential effective means to promote healthy food is using food advertising that appeals to consumers and enhances their taste perceptions of healthy food. 

Recent research on advertising effectiveness has focused on sensory marketing as an efficient way to engage consumers [13,14,15,16]. Sensory marketing engages the consumers’ senses and affects their perception, judgment and behavior [15]. Applied to food, sensory food advertisements are used to create sensory triggers that affect the perceived quality of an abstract food attribute like its taste, smell, or shape [15]. According to Elder and Krishna [17], multisensory advertising for food can enhance taste perceptions of the food. These authors argue that ads referring to multiple senses (multiple-sense ads) for indulgent foods evoke more positive sensory thoughts about the food (e.g., “I like the crunchy texture of potato chips”) compared to ads mentioning taste only (single-sense ads), and hence these positive sensory thoughts optimize perceived taste of the food.

However, this previous research only investigated the effects of sensory advertising for unhealthy food. Considering the increasing obesity rates and the human tendency to favor unhealthy food over healthy food [18,19], an important objective today is to promote the consumption of healthy food by use of sensory advertising [10,11]. Additionally, Elder and Krishna [17] only examined the effects of sensory advertising with verbal references to the senses (i.e., slogans), by using wording that evokes sensory thoughts. Today’s consumer environment, however, is mainly dominated by visual stimuli that are omnipresent in online advertising and social media, but also in offline marketing channels [20,21,22]. As food selection and consumption is—besides the other senses—primarily guided by our sight [23,24], it is worth investigating whether sensory advertising for healthy food with visual references to the senses (e.g., advertising pictures) has similar effects as sensory advertising for healthy food with verbal references the senses (e.g., ad slogans). Furthermore, Elder and Krishna [17] limit their research to the effect of multi-sensory versus single-sensory advertising on taste perceptions. Even though perceived taste is an important consumer judgment, it is also valuable to assess whether sensory advertising for healthy food affects advertising effectiveness, like consumers’ attitudes toward advertising and behavioral intentions, which can impact consumer purchase behavior [25,26].

Addressing these three limitations, we, therefore, investigate for healthy food advertising, whether (1) verbal and (2) visual sensory advertising influence both taste perceptions and (3) advertising effectiveness. Building on sensory perception literature and food marketing research, we examine whether the use of multiple senses in food advertising, shown to be effective in promoting unhealthy food, is also effective to promote healthy food. Essentially, we expect an opposite effect for healthy food. Whereas unhealthy food is considered to be tempting, this does not hold to the same extent for healthy food [4], for which we might need less persuading. Advertisements for healthy food referring to multiple senses, therefore, might raise the chance of evoking critical thinking and negative thoughts. Consequently, these negative thoughts can reduce taste expectations and consumer attitudes. We argue that referring to a single sense compared to multiple senses in healthy food advertising reduces the chance of evoking negative thoughts and, as such, increases consumer attitudes.

This research contributes to the literature in three ways. First, while multiple-sense ads for unhealthy food were shown to be more effective than single-sense ads, we find that, for healthy food, single-sense ads increase taste perceptions and advertising effectiveness compared to multiple-sense ads. Second, we show that this effect arises because of a different underlying process. Although for unhealthy food the effect of single-sense versus multiple-sense advertising can be explained by the generation of ‘positive thoughts’, we find that in the case of healthy food, ‘negative thoughts’ are at play. Specifically, single-sense ads evoke fewer negative thoughts than multiple-sense ads, which mediates the effect of single-sense versus multiple-sense ads on taste perceptions and advertising effectiveness. Third, we show that these effects occur not only for verbal ads but, more importantly, also for visual ads, which are omnipresent today. 

To the best of our knowledge, this research is the first to investigate the effectiveness of sensory advertising for healthy food promotion. The results from our studies point to the possibility of using the right kind of sensory advertising for healthy food to create favorable food product evaluations. As such, we provide specific practical implications for food marketers, ad executives, and public policy. Applying our findings to advertising campaigns promoting healthy food can be a valuable step in the right direction to decrease obesity rates and instead increase healthy food consumption.

### 1.1. Sensory Advertising for Healthy versus Unhealthy Food

Food advertising is omnipresent in our consumer lives. Especially in today’s digital environment, we are constantly exposed to food cues. Think about how lots of food ads pop up on your internet browser after Googling a recipe. While food advertising is typically used to increase interest in the food or an intention to buy or consume the food, it usually cannot easily affect expected taste perceptions [15]. However, taste plays a primary role in directing consumers’ food choice [27]. Indeed, the food quality properties that the majority of consumers value the most are taste, freshness, and nutritional value [28]. In making consumption decisions, consumers consider both intrinsic food attributes, which are an integral part of a product (e.g., food composition), and extrinsic cues, which are immaterial components that exert no influence on the physical product when changed (e.g., verbal descriptions or images of the food) [5]. More specifically, taste perceptions are derived from multiple intrinsic sensory components, including smell, sound, touch, and vision [17]. Although these intrinsic food attributes mainly guide taste perceptions, extrinsic cues can also shape perceived taste of food. For instance, taste perceptions can be susceptible to sensory cues in food advertising [15]. This is why food advertising frequently mentions the taste of food [16,17]. Think, for example, about the company name “Freakin’ Delicious Cookies Inc.”.

Nonetheless, even though we are constantly exposed to food stimuli and eat frequently, we are not very good at accurately judging the taste of food [15,17]. This is because consuming food involves a multisensory taste experience, as taste relies heavily on input from the other senses. For example, imagine yourself having a stuffy nose and not being able to taste appropriately because you lack the ability to smell. Or imagine how hard it can be, when being blindfolded, to guess what you are eating. Taste is hard to define when your sight is blocked. These examples illustrate how every sense has a role in affecting taste [15,17]. Thus, taste is derived from multiple sensory cues, including smell, vision, sound, and touch. In other words, every single taste is a combination of all our five senses [15].

This is why, more recently, advertising researchers have focused on sensory marketing, which is defined as marketing that engages the consumers’ senses and affects their perception, judgment and behavior [13,14,15]. Given the daily overload of advertisements that consumers are confronted with, it seems that unconscious triggers, like those appealing to the basic senses, may be a more efficient way to appeal to consumers [15]. Applied to food, sensory food advertisements can be used to create sensory triggers that affect the perceived quality of an abstract food attribute like its taste, smell or shape [15].

According to Elder and Krishna [17], advertising for food can affect taste perceptions by affecting sensory cognitions. These authors show that an advertisement emphasizing multiple senses (e.g., taste, touch and smell) results in better taste perception than an advertisement emphasizing only taste. They show that this effect works through sensory stimulation [15]. Specifically, they found that multiple-sense ads for indulgent foods evoke more positive sensory thoughts about the foods compared to ads mentioning taste only, and hence these positive thoughts optimize perceived taste. Indulgent or tempting foods are generally experienced as pleasant and tasty [29]. As such, ads that mention different aspects of this pleasant experience, by focusing on multiple different senses (such as taste, smell and texture), should generally evoke more positive sensory thoughts and thus be more effective compared to ads that mention only one sense (such as taste). Effectively, advertising approaches commonly used to promote indulgent foods utilize hedonic consumption themes of sensory stimulation, pleasure, fun, and excitement [30,31,32].

An important limitation of the research conducted by Elder and Krishna [17] is that the authors only investigated unhealthy tempting food—the food stimuli used in their studies were potato chips, popcorn, and chewing gum [17]. At first sight, the findings by Elder and Krishna [17] could suggest that multiple-sense ads can also promote healthy food. However, previous research shows that healthy food advertising and unhealthy food advertising often use different advertising techniques [33] and also call for different advertising techniques in order to be effective [34].

What is crucial in (food) advertising is that the advertising technique (e.g., type of slogan, type of spokesperson, etc.) matches the specific product type [35,36]. In the case of healthy and unhealthy food, multiple core characteristics discern these two product types. The aforementioned definitions of healthy food as a virtue—a product that is not very tempting immediately but is advantageous in the long run (i.e., something less likely to cause regret)—and of unhealthy food as a vice—a tempting product which consumption does not imply long-term benefits (i.e., something truly desired but at the same time guilt-inducing)—reveal some of the crucial differences between the two food categories [4]. Essentially, this pertains to the level of food temptation and the extent to which the food induces feelings of guilt. We believe these differences—among others—provide a potential explanation for why multiple-sense ads might not be equally effective to promote healthy food compared to unhealthy food. 

We reason that in the case of the promotion of unhealthy food—and thus tempting but also guilt-inducing food—consumers like to be persuaded. It is as if the persuasive claims take away the conflict consumers experience when they want to indulge [37]. The higher the persuasion intent, the more consumers are motivated to consume the food product. We assume this is the reason why multiple-sense ads referring to multiple senses or bringing up multiple arguments increase the taste perceptions of unhealthy food compared to single-sense ads that refer to only one sense or argument [17]. The amount of positive thoughts that persuade consumers to consume the unhealthy and tempting food product mediates this effect [17]. In short, overselling does no harm when promoting unhealthy food. 

Importantly, however, when promoting healthy food, we assume consumers perceive the persuasion intent as less encouraging and potentially even as discouraging to consume the food. This is because temptations or feelings of guilt are less at play when consumers are presented with healthy food [4]. Consequently, consumers have a lower yearning desire to be persuaded and are more prudent towards the advertising claims [38]. Following this reasoning, we expect that a (too) high level of perceived persuasion intent in healthy food advertising (e.g., referring to multiple senses in one advertisement) can backfire and be experienced as ‘overselling’. Therefore, we argue that multiple-sense advertising for healthy food initiates critical processing and, therefore, can evoke negative thoughts towards the food product (e.g., regarding its taste) and the advertisement, compared to when consumers perceive a lower level of persuasion intent, such as in the case of a single-sense advertisement. This assumption is strengthened by previous research showing a preference of a single (nutrition) claim over multiple simultaneous (nutrition) claims [39]. 

Building on this framework, we hypothesize that when consumers see a single-sense advertisement for healthy food, they will evoke less critical processing and negative thoughts compared to when they see a multiple-sense advertisement. This lower level of negative thoughts explains why single-sense ads lead to higher taste perceptions than multiple-sense ads.

Furthermore, contrasting to Elder and Krishna [17], we do not only examine the effect of sensory advertising on taste perceptions, but we also investigate the advertising effectiveness of using single-sense versus multiple-sense references in healthy food advertising. In advertising studies, three traditional measures of advertising effectiveness are commonly used: attitude toward the ad (Aad), attitude toward the advertised brand (Ab), and purchase intention (PI). Therefore, the current research adopts these measures to assess consumers’ responses to sensory advertisements for healthy versus unhealthy food. Findings from previous studies strongly indicate that these three constructs are related and impact consumer purchase behavior [25,26].

To summarize, we reason that single-sense ads for healthy food will increase both taste perceptions and advertising effectiveness compared to multiple-sense ads. We argue that this effect arises because single-sense ads for healthy food evoke fewer negative thoughts than multiple-sense ads, which will mediate the effect of single-sense versus multiple-sense ads on taste perceptions and advertising effectiveness.

### 1.2. Verbal and Visual Sensory Food Advertising

Sensory advertising for food can use verbal references to the senses (e.g., slogans), by using wording that refers to the taste, smell, vision, sound, and touch of food [17]. However, referring to the senses in sensory food advertising can also be done by using visual references, with pictures or visuals that display certain senses (e.g., holding, smelling, or biting an apple). Nowadays, visual stimuli are omnipresent in our society, and especially in the consumer consumption environment. Food advertising in magazines, television, digital marketing, and social media constantly uses visual cues of both healthy and unhealthy food (e.g., Red Bull’s social media videos or Weight Watcher’s motivational content on social media). Moreover, as consumers’ food selection and consumption are—besides the other senses—primarily guided by the visual system [23,24], the increasing use of visual food stimuli strengthens the importance of using visual cues in food advertising. 

Prior research indeed shows that besides verbal ads, visual ads are also effective [21,22,40]. Visual aesthetics are recognized as key determinants of advertising effectiveness. This is because visual stimuli, such as ad pictures, can evoke mental imagery or simulation, similar to verbal stimuli, such as slogans [16]. People who see a stimulus may spontaneously form a visual image of it, just like reading about a stimulus [41,42,43]. Consequently, mental imagery about a stimulus can drive cognition, and this imagery can evoke thoughts about the stimulus [15,44]. In other words, imagery processing evokes the consumer’s use of sensory perceptions which may direct attention towards the possible outcomes and experiences of the stimulus [45]. Several neuroimaging studies provide evidence for such mental simulation whereby conceptual processing of sensory perceptions leads to neural activation of corresponding regions of the brain [15]. For instance, pictures of food lead to similar neural activation patterns in the orbitofrontal cortex as verbal descriptions, and additionally they activate areas associated with reward [46].

Therefore, we argue that the effects of single-sense ads for healthy food occur when using verbal references to senses (i.e., ad slogans), but also in the case of visual references (i.e., advertising pictures). By investigating the effects of visual sensory advertising, we aim to contribute, step-by-step, to filling the void about the do’s and don’ts for visual food marketing today.

### 1.3. Research Aims and Hypotheses

In two laboratory experiments, we investigate how sensory advertising can promote healthy food. We propose that single-sense ads for healthy food increase taste perceptions and advertising effectiveness compared to multiple-sense ads. We argue that this effect arises because single-sense ads for healthy food evoke fewer negative thoughts than multiple-sense ads, which will mediate the effect of single-sense versus multiple-sense ads on both taste perceptions and advertising effectiveness. Additionally, we examine whether these effects occur for verbal (i.e., ad slogans) and visual (i.e., advertising pictures) sensory advertisements for healthy food. Because both verbal and visual stimuli can evoke sensory thoughts, we investigate the effect of sensory food advertising using verbal references (Study 1), and visual references to the senses (Study 2). More formally, we propose that:

**Hypothesis** **1** **(H1).** *For healthy food, taste perceptions and advertising effectiveness will be higher for ads that refer to taste only (single-sense ads), compared to ads referring to multiple senses (multiple-sense ads).*

**Hypothesis** **2** **(H2).** *For healthy food, single-sense ads will evoke fewer negative thoughts than multiple-sense ads.*

**Hypothesis** **3** **(H3).** *The effect of single-sense versus multiple-sense ads for healthy food on taste perceptions and advertising effectiveness will be mediated by negative thoughts.*

## 2. Study 1: Verbal Sensory Advertising for Healthy versus Unhealthy Food

### 2.1. Study Overview

In this study, we test whether a single-sense ad slogan for healthy food results in higher taste perceptions and advertising effectiveness (H1), and in fewer negative thoughts (H2), compared to a multiple-sense ad slogan. We also test whether these negative thoughts mediate the effect of single-sense versus multiple-sense slogans on taste perceptions and advertising effectiveness (H3). We use verbal ad slogans, with one focusing explicitly on taste, and the other on the general sensory experience, similar to Elder and Krishna [17]. In this study, we assess negative thoughts with a metric scale. For the robustness of the mediation effect, this measurement differs from the one in Study 2, where we use an ‘open-thought listing task’. To rule out any effect of negative thoughts in the case of unhealthy food advertising, we adopted two additional conditions—an unhealthy single-sense ad slogan condition and an unhealthy multiple-sense ad slogan condition.

### 2.2. Participants

A total of 158 university students (36% men, *M_age_* = 23 years; *SD* = 6.88) participated in this study. All participants were granted one credit for completing the experiment, which was included in a 50 min session of multiple unrelated studies. All participants gave their informed consent for inclusion before they participated in the study. Seventeen participants were removed from the dataset due to not being allowed to eat the experimental food product (*n* = 13), or due to experiencing problems during the study (*n* = 4). Our final sample included 141 participants. They were randomly assigned to one of four between-subjects conditions: the healthy single-sense ad slogan condition (*n* = 37), the unhealthy single-sense ad slogan condition (*n* = 35), the healthy multiple-sense ad slogan condition (*n* = 31), or the unhealthy multiple-sense ad slogan condition (*n* = 38). None of the participants was familiar with the experimental slogan and brand. This study was conducted in accordance with the Declaration of Helsinki for Research involving Human Subjects and received approval from the Ethics Committee of the Faculty of Economics and Business Administration, Ghent University.

### 2.3. Stimuli and Procedure

After being welcomed to the lab session, participants were asked to fill out their sex and age. Next, they were informed that this study was about a new brand of cherry tomatoes (i.e., healthy food condition) or potato chips (i.e., unhealthy food condition), and that they would be asked to evaluate the ad slogan and taste of the advertised product. Participants then saw a single-sense ad slogan or a multiple-sense ad slogan promoting either cherry tomatoes or potato chips, depending on the condition they were assigned to. We chose the same fictitious brand name “Valens” for the two food products. For the potato chips, the ad slogans were as follows: (1) unhealthy single-sense condition: “*Our potato chips deliver the taste you crave. From the first bite on you’ll savor the rich barbecue flavor and enjoy the nice bell pepper taste. Our potato chips are the perfect choice for your snacking*”; (2) unhealthy multiple-sense condition: “*Our potato chips deliver the taste you crave. From the first bite on you’ll savor the right barbecue smell and enjoy the delicious crunch texture. Our potato chips are the perfect choice for your snacking*”. These two slogans were based on the ad slogans used by Elder and Krishna [17]. The ad slogans for the cherry tomatoes were analogous to the single-sense and multiple-sense ad slogan for the potato chips: (3) healthy single-sense condition: “*Our cherry tomatoes deliver the taste you crave. From the first bite you’ll savor the sweet touch and enjoy the nice complete taste. Our cherry tomatoes are the perfect choice for your snacking*”; (4) healthy multiple-sense condition: “*Our cherry tomatoes deliver the taste you crave. From the first bite you’ll enjoy the juicy texture and savor the sweet flavor. Our cherry tomatoes are the perfect choice for your snacking*”. After exposure to the ad slogan, participants were asked to go to the lab instructor and ask for a plate of cherry tomatoes (potato chips). We used the exact same plates to present the cherry tomatoes and the potato chips. Participants in the healthy food condition received 3 cherry tomatoes, and participants in the unhealthy food condition received the same amount of chips (±100 grams). After having tasted the food product, participants proceeded to fill out the questionnaire.

### 2.4. Measurements and Reliability

We first measured negative thoughts elicited by the ad slogan with a one-item 7-point Likert scale. Participants were asked: “*When you consider your thoughts while reading the slogan, to what extent did you have negative thoughts?*” (1 = Absolutely not, 7 = Absolutely yes). In addition, we included the item “*I thought about something else than the slogan*” (1 = Absolutely not, 7 = Absolutely yes) as a covariate, indicating how much participants were involved in the task. Second, taste perceptions of the food product were measured in a similar way as Elder and Krishna [17]. Participants were asked on a 7-point Likert scale their opinion about (1) “*The quality of the product*” (1 = Very poor quality, 7 = Very good quality), (2) “*The overall taste*” (1 = Very poor taste, 7 = Very good taste), and (3) “*How delicious the product was*” (1 = Not at all delicious, 7 = Very delicious). Taste perception measures (i.e., the quality of the product, overall taste, and how delicious the product was) were combined into a new variable, taste perceptions (*α* = 0.91), by averaging the responses to the three items. Third, we measured Aad on a 7-point bipolar scale with three items: (1) “*This is a bad (good) ad*”, (2) “*This ad is unattractive (attractive)*”, and (3) “*My opinion about this ad is negative (positive)*”. These three items were combined into a new variable, Aad (*α* = 0.89), by averaging the responses. Fourth, we measured Ab on a 7-point bipolar scale with three items: (1) “*I have a negative (positive) feeling about Valens tomatoes (potato chips)*”, (2) “*I do not love (love) Valens tomatoes (potato chips)*”, and (3) “*Valens is not an attractive brand (is an attractive brand)*”. These three items were combined into a new variable, Ab (*α* = 0.89), by averaging the responses. Finally, we measured PI on a 7-point Likert scale with two items: (1) “*It seems a good idea to buy Valens tomatoes (potato chips)*”, (2) “*If I would buy tomatoes (potato chips), I would buy Valens tomatoes (potato chips)*” (1 = Totally disagree, 7 = Totally agree*).* These two items were combined into a new variable, PI (*r* = 0.735, *p* < 0.001), by averaging the responses.

### 2.5. Data Analysis

To compare the taste perceptions, ad effectiveness, and negative thoughts between the single-sense and multiple-sense ad slogans, and between the healthy and unhealthy food ad slogans, we conducted a Multivariate Analysis of Covariance (MANCOVA) and a two-way Univariate Analysis of Covariance (ANCOVA) with, respectively, ad type (i.e., single-sense ad slogan vs. multiple-sense ad slogan) and product type (i.e., healthy food product vs. unhealthy food product) as independent variables, negative thoughts, taste perceptions, and ad (slogan) effectiveness (i.e., Aad, Ab, and PI) as dependent measures, and ‘thinking about something else than the slogan’ (defined as ‘other thoughts’) as covariate (testing H1 and H2). In addition, we ran four moderated mediation analyses via the PROCESS macro of Preacher and Hayes. This is an observed variable OLS and logistic regression path analysis modeling tool [47] which allows us to test whether negative thoughts mediate the effect of ad type on respectively taste perceptions, Aad, Ab, and PI for the healthy food product (testing H3). In these analyses, ad type served as independent variable, food product type as moderating variable, negative thoughts as mediator, taste perceptions and ad effectiveness as dependent measures, and other thoughts as covariate. These analyses also enable us to verify that negative thoughts do not mediate the effect for the unhealthy food product condition. All data were analyzed using IBM SPSS Statistics 25 (IBM corp., Armonk, NY, USA).

### 2.6. Results and Discussion

#### 2.6.1. Main Effects Analyses

The two-way MANCOVA shows a marginally significant total direct effect of ad type on taste perceptions (*F*(1, 136) = 2.96, *p* = 0.088), with the single-sense ad slogan (*M* = 7.15, *SD* = 1.60) leading to more positive taste perceptions than the multiple-sense ad slogan (*M* = 6.68, *SD* = 1.61). No significant effect of ad type on Aad (*F*(1, 136) = 0.00, *p* = 0.098), Ab (*F*(1, 136) = 0.69, *p* = 0.407), and PI (*F*(1, 136) = 0.58, *p* = 447) was found. The effect of product type on Aad (*F*(1, 136) = 7.82, *p* = 0.006), and on PI (*F*(1, 136) = 4.50, *p* = 0.036) was significant. Specifically, Aad and PI were higher for the healthy food product (*M_Aad_* = 4.99, *SD* = 1.38; *M_PI_* = 4.90, *SD* = 1.39), compared to the unhealthy food product (*M_Aad_* = 4.34, *SD* = 1.38; *M_PI_* = 4.40, *SD* = 1.39). The effect of product type on taste perceptions (*F*(1, 136) = 0.05, *p* = 0.833), and on Ab (*F*(1, 136) = 1.07, *p* = 0.302) was not significant. The interaction effect of ad type and product type on taste perceptions (*F*(1, 136) = 0.62, *p* = 0.431), Ab (*F*(1, 136) = 1.87, *p* = 0.173), and PI (*F*(1, 136) = 0.04, *p* = 0.834) was not significant either. More detailed analyses can help unravel these results. 

Simple contrasts show that no significant effects of ad type within the unhealthy food product are found for taste perception (*F*(1, 136) = 0.46, p = 0.501), Aad (*F*(1, 136) = 2.07, *p* = 0.153), Ab (*F*(1, 136) = 0.15, *p* = 0.703), and PI (*F*(1, 136) = 0.16, *p* = 0.690). In the case of the healthy food product, there is a marginally significant effect of ad type on taste perceptions (*F*(1, 136) = 3.05, *p* = 0.083), with the single-sense ad slogan (*M* = 7.22; *SD* = 1.61) evoking more positive taste perceptions compared to the multiple-sense ad slogan (*M* = 6.54; *SD* = 1.60). The effect of other thoughts as covariate on taste perceptions was significant (*F*(1, 136) = 4.36, *p* = 0.039). No significant effects were found for Aad (*F*(1, 136) = 1.84, *p* = 0.177), Ab (*F*(1, 136) = 2.34, *ns*) and PI (*F*(1, 136) = 0.46, *p* = 0.499). As such, these results partly confirm H1 on taste perceptions, on a 90% significance level. Specifically, for healthy food, taste perceptions are higher for the single-sense ad slogan compared to the multiple-sense ad slogan. However, to draw final conclusions about the relationship between ad type and taste perceptions and ad effectiveness, further mediation analyses were necessary, as indirect mediation only can still be at hand regarding taste perceptions and ad effectiveness [48].

**Figure 1:**
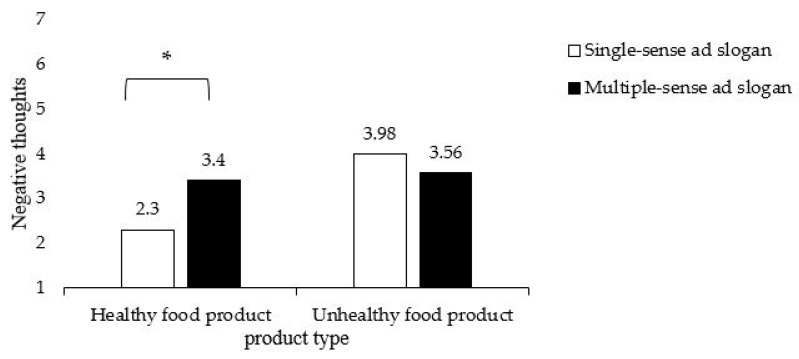
Results of Study 1. The interaction of ad type and product type on negative thoughts. Note: * significant at *p* < 0.05.

In more detailed analyses, using a two-way ANCOVA, we find that the main effect of ad type on negative thoughts is not significant (*F*(1, 136) = 0.96, *p* = 0.329), whereas the main effect of product type on negative thoughts is significant (*F*(1, 136) = 7.30, *p* = 0.008). Specifically, potato chips (*M* = 3.77, *SD* = 2.02) lead to more negative thoughts compared to cherry tomatoes (*M* = 2.85, *SD* = 2.03). More importantly, the interaction effect of ad type and product type on negative thoughts is significant (*F*(1, 136) = 4.97, *p* = 0.027; Figure 1). Other thoughts as covariate significantly affected this interaction effect (*F*(1, 136) = 6.08, *p* = 0.015). Simple contrast effects show that there is no effect of ad type on negative thoughts for the unhealthy food product (*M*_single_ = 3.98, *SD* = 2.02; *M*_multiple_ = 3.56, *SD* = 2.03; *F*(1, 136) = 0.80, *p* = 0.374), whereas there is a significant effect of ad type on negative thoughts for the healthy food product (*F*(1, 136) = 4.97, *p* = 0.027). In the case of healthy food, the single-sense ad slogan leads to fewer negative thoughts (*M* = 2.30, *SD* = 2.03), compared to the multiple-sense ad slogan (*M* = 3.40, *SD* = 2.02). This confirms H2 on a 95% significance level. For healthy food, fewer negative thoughts are evoked by the single-sense ad slogan compared to the multiple-sense ad slogan.

#### 2.6.2. Moderated Mediation Analyses 

**Figure 2:**
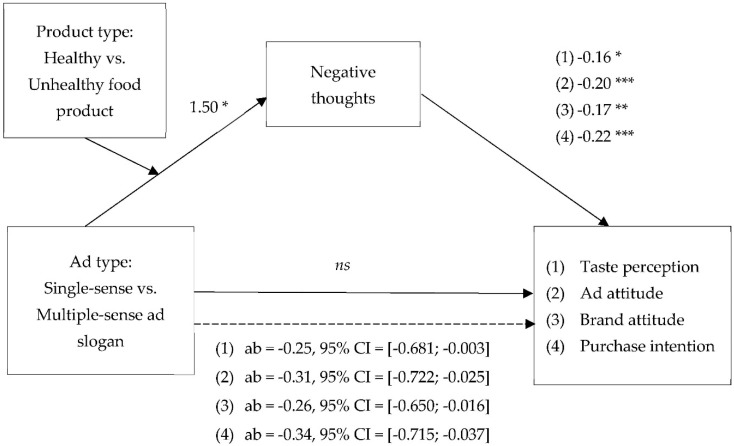
Results of Study 1. The effect of ad type and product type on ad effectiveness is mediated by negative thoughts. Note: Other thoughts (*t*(136) = 2.46, *p* = 0.015); *** significant at *p* < 0.001; ** significant at *p* < 0.01; * significant at *p* < 0.05; ab = indirect effect; CI = Confidence Interval.

According to the moderated mediation analyses (model 7; Figure 2) [47], the interaction effects of ad type (i.e., single-sense ad slogan vs. multiple-sense ad slogan) and product type (i.e., healthy food product vs. unhealthy food product) on taste perceptions and on ad effectiveness are mediated by negative thoughts. The analyses, with 10,000 bootstraps and 95% bias-corrected intervals (CIs), reveal a significant moderated mediation for taste perceptions (ab = −0.24, *SE* = 2.16, 95% CI = [−0.691; −0.003]), Aad (ab = −0.31, *SE* = 2.14, 95% CI = [−0.722; −0.025]), Ab (ab = −0.26, *SE* = 1.90, 95% CI = [−0.650; −0.016]), and PI (ab = −0.34, *SE* = 2.02, 95% CI = [−0.715; −0.036]). Furthermore, the moderated mediation analysis shows that the effect of negative thoughts on taste perceptions is significant (*t*(136) = −2.56, *p* = 0.011), and that the remaining direct effect is not significant (*t*(137) = −1.50, *p* = 0.137). The effects of negative thoughts on Aad (*t*(137) = −3.67, *p* < 0.001), Ab (*t*(137) = −3.38, *p* = 0.001) and PI (*t*(137) = −4.13, *p* < 0.001) are significant too, and the remaining direct effects are not significant, respectively: Aad (*t*(137) = 0.24, *p* = 0.808), Ab (*t*(137) = −0.58, *p* = 0.563) and PI (*t*(137) = −0.56, *p* = 0.577).

To conclude, the interaction effect of ad type and product type on taste perceptions is fully mediated by negative thoughts. These results confirm H3. The effect of single-sense versus multiple-sense ad slogans for healthy food on taste perceptions and advertising effectiveness is mediated by the negative thoughts evoked by the ad slogan.

## 3. Study 2: Visual Sensory Advertising for Healthy Food

### 3.1. Study Overview

Study 2 aims to extend the findings from Study 1. Study 2 differs from Study 1 in four ways. First, Study 2 only focuses on healthy food, as our primary research purpose is to test the effectiveness of sensory advertising for healthy food. Second, while Study 1 used verbal references to the senses (i.e., ad slogans), this study uses visual references, by using pictures in an advertisement. Third, we use a different measurement for the underlying process of negative thoughts evoked by the visual sensory ads as using multiple measurements for the same constructs over different studies provides additional robustness. While Study 1 used a metric scale to assess negative thoughts, this study used an open-thought listing task, similar to the task used by Elder and Krishna [17] to measure positive sensory thoughts. Fourth, we did not include an actual taste test in this study for reasons of external validity. Marketing promotions often include advertising cues that are presented in environments where the advertised product is not immediately purchasable or ready to taste (e.g., printed advertisements, street campaigns, etc.). 

### 3.2. Pretest 

First, we pretested whether the single-sense and multiple-sense visual ads promoting healthy food were equally perceived as professional advertisements.

#### 3.2.1. Pretest Participants

A total of 76 U.S. respondents from Amazon’s Mechanical Turk (MTurk) platform (54% men, *M_age_* = 33 years; *SD* = 10.24) participated in this pretest. Participants received 30 cents for completing the study, which took about 3 min. All participants gave their informed consent for inclusion before they participated in the study. According to Huff and Tingley [49], MTurk participants represent diverse industries and geographic locations (90% urban areas) as effectively as professionally obtained samples do, and so these samples offer valid input to make inferences about broader populations of interest. Participants were randomly assigned to one of two between-subject conditions: the healthy single-sense advertising picture condition (*n* = 37), or the healthy multiple-sense advertising picture condition (*n* = 39). None of the participants were familiar with the advertisement picture. This study was conducted in accordance with the Declaration of Helsinki for Research involving Human Subjects and received approval from the Ethics Committee of the Faculty of Economics and Business Administration, Ghent University.

#### 3.2.2. Pretest Stimuli and Procedure

**Figure 3:**
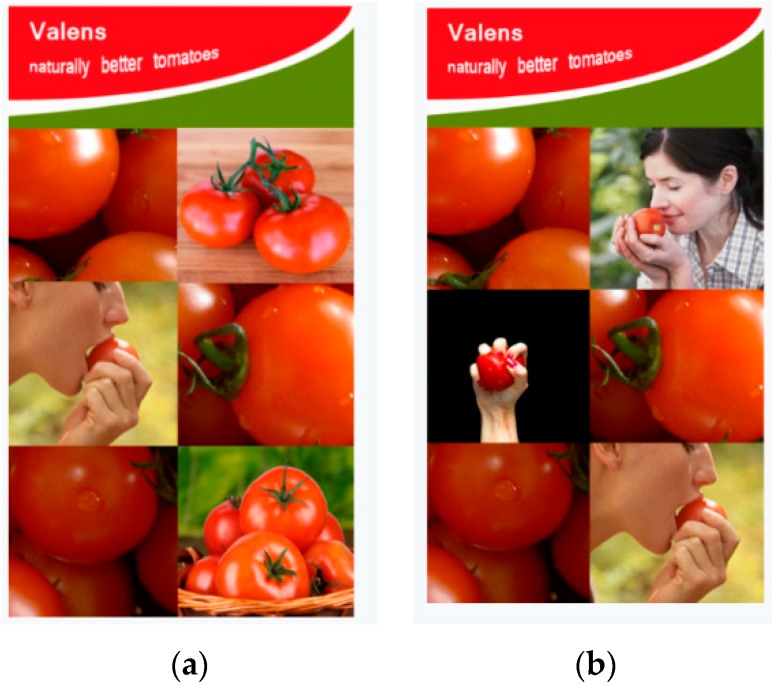
Experimental stimuli used in Study 2: (**a**) Single-sense ad; (**b**) Multiple-sense ad.

After being welcomed to the online session, participants read that the researchers were interested in their opinions about a specific visual advertisement of a new brand of tomatoes, ‘Valens’. Next, participants saw one advertisement (i.e., single-sense or multiple-sense visual ad), depending on the condition they were assigned to (Figure 3). In both conditions, the promoted product was tomatoes. We made sure that the pictures looked as similar as possible (e.g., use of colors, number of pictures, etc.), except for the representation of only one sense (i.e., taste) or multiple senses (i.e., taste, smell, and touch). After exposure to the visual advertisement, participants filled out the questionnaire.

#### 3.2.3. Pretest Measurements and Reliability

Three items measured whether the visual advertisements were perceived as professional. Participants indicated to what extent: (1) “*The ad looks unrealistic (realistic)*”, (2) “*The ad is atypical (typical)*”, and (3) “*This type of ad is unexpected (common) for promoting this product*”, on 7-point bipolar scales. 

#### 3.2.4. Data Analysis

To compare the perceived professionalism of the advertisements between the single-sense and multiple-sense ad, a one-way Analysis of Variance (ANOVA) was conducted. All data were analyzed using IBM SPSS Statistics 25 (IBM Corp., Armonk, NY, USA).

#### 3.2.5. Results and Discussion

The results of a one-way ANOVA show a marginally significant effect of ad type on how professional the ads look (*F*(1, 74) = 2.96, *p* = 0.090). The single-sense ad was perceived as more common for promoting the product (*M* = 4.16; *SD* = 1.84), than the multiple-sense ad (*M* = 3.44; *SD* = 1.84). The effect of ad type on looking realistic (*F*(1, 74) = 0.05, *p* = 0.830), and on looking typical (*F*(1, 74) = 2.33, *p* = 0.130) was not significant. As these results indicate, there are no large differences between the single-sense visual ad and the multiple-sense visual ad on perceived professionalism, and so we decided to use these ads as experimental stimuli for the main study.

### 3.3. Main Study

#### 3.3.1. Participants

A total of 99 university students (43% men; *M_age_* = 23 years; *SD* = 5.04) participated in this study. All participants were granted one credit for completing the experiment, which was included in a 50 min session of multiple studies. All participants gave their informed consent for inclusion before they participated in the study. We excluded 15 participants from the dataset as they were not allowed to eat the experimental food product (*n* = 7), or due to experiencing problems during the study (*n* = 4), or missing data (*n* = 5). Our final sample included 83 participants. Participants were randomly allocated into the single-sense visual ad condition (*n* = 40), or the multiple-sense visual ad condition (*n* = 43). None of the participants were familiar with the advertisement picture and brand. This study was conducted in accordance with the Declaration of Helsinki for Research involving Human Subjects and received approval from the Ethics Committee of the Faculty of Economics and Business Administration, Ghent University.

#### 3.3.2. Stimuli and Procedure

The procedure was similar to Study 1. After being welcomed to the lab session, participants were informed that this study was about a new brand of tomatoes, ‘Valens’, and that they would be asked to evaluate the advertisement. They started with filling out their sex and age. Next, participants saw either the single-sense visual ad or the multiple-sense visual ad, both promoting tomatoes via pictures, depending on the condition they were assigned to (Figure 3). After exposure to the advertisement, participants proceeded to fill out the questionnaire.

#### 3.3.3. Measurements and Reliability 

First, we measured negative thoughts with an open-thought listing task [17]. Participants were asked to list everything they were thinking after viewing the visual advertisement [50]. Participants’ thoughts were coded in the same way as in Elder and Krishna [17]. We coded for valence as positive (e.g., “*The tomatoes look tasty*”), negative (e.g., “*I do not like water drops on the tomatoes*”), and neutral (e.g., “*I often eat tomatoes at my parents’ place*”). The thoughts were further coded by content as being primarily sensory (e.g., “*I would like to touch the tomato*”), brand related (e.g., “*I don’t know the brand Valens*”), or ad related (e.g., “*I never saw somebody smelling a tomato*”). Similar to Study 1, we also coded for ‘other thoughts’, defined as to what extent participants were not thinking about the advertisement.

Following the open-thought listing task, we measured taste perceptions and ad effectiveness. Similar to Study 1, we asked participants their perception about the taste of the food product on a 7-point Likert scale with two items: (1) “*The overall taste*” (1 = Very poor taste, 7 = Very good taste), and (2) “*How delicious the tomatoes were*” (1 = Not at all delicious, 7 = Very delicious). As this study focuses only on healthy food products, and as the core aspect of healthy food is its healthiness, we asked participants to rate the perceived overall healthiness of the product instead of product quality. Perceived overall healthiness of the product was measured on a 7-point Likert scale (1 = Very unhealthy, 7 = Very healthy). These three measures (i.e., perceived overall taste, perceived deliciousness, and perceived overall healthiness) were combined into a new variable, taste perceptions (*α* = 0.89), by averaging the responses. Next, Aad and Ab were measured with the same scales as in Study 1. We measured Aad using a 7-point bipolar scale with three items: (1) “*This is a bad (good) ad*”, (2) “*This ad is unattractive (attractive)*”, and (3) “*My opinion about this ad is negative (positive).*” These three items were combined into a new variable, Aad (*α* = 0.96), by averaging the responses. Ab was measured on a 7-point bipolar scale with three items: (1) “*I have a negative (positive) feeling about Valens tomatoes*”, (2) “*I do not love (love) Valens tomatoes*”, and (3) “*Valens is not an attractive brand (is an attractive brand).*” These three items were combined into a new variable, Ab (*α* = 0.91), by averaging the responses. Finally, we measured PI on a 7-point Likert scale with three items: (1) “*It seems a good idea to buy Valens tomatoes*”, (2) “*If I would buy tomatoes, I would buy Valens tomatoes*”, and (3) “*From now on I will pay more attention to Valens tomatoes*” (1 = Totally disagree, 7 = Totally agree). These three items were combined into a new variable, PI (*α* = 0.82), by averaging the responses. 

#### 3.3.4. Data Analysis

To test H1, we ran a Multivariate Analysis of Covariance (MANCOVA), with ad type (i.e., single-sense ad vs. multiple-sense visual ad) as independent variable, taste perceptions, Aad, Ab, and PI as dependent variables, and other thoughts as covariate. To compare the number of negative thoughts between the single-sense and multiple-sense ad (testing H2), we ran a one-way Multivariate Analysis of Variance (MANOVA) with ad type (i.e., single-sense visual ad vs. multiple-sense visual ad) as independent variable, and number of thoughts as dependent variable. To test H3, whether negative thoughts mediate the effect of ad type on taste perceptions and ad effectiveness (i.e., Aad, Ab, and PI), we conducted four mediation analyses via PROCESS [47]. All data were analyzed using IBM SPSS Statistics 25 (IBM corp., Armonk, NY, USA).

#### 3.3.5. Results and Discussion

The MANCOVA shows no significant effect of ad type on taste perceptions (*F*(1, 80) = 2.28, *p* = 0.135*)*. However, there is a significant effect of ad type on Aad (*F*(1, 80) = 4.94, *p* = 0.029): the single-sense visual ad leads to a higher Aad (*M* = 4.97, *SD* = 1.69), compared to the multiple-sense visual ad (*M* = 4.14, *SD* = 1.69). Further, we found a marginally significant effect for Ab (*F*(1, 80) = 2.97, *p* = 0.089), and for PI (*F*(1, 80) = 3.65, *p* = 0.060), with the single-sense visual ad leading to a higher Ab (*M* = 4.83, *SD* = 1.36), and a higher PI (*M* = 4.36, *SD* = 1.17), compared to the multiple-sense visual ad (*M* = 4.32, *SD* = 1.35 for Ab, and *M* = 3.87, *SD* = 1.17 for PI). Other thoughts as covariate was marginally significantly related to all dependent variables at the 90% significant level (*F*(1, 80) > 2.96, *p* < 0.090). These results partly confirm H1 for advertising effectiveness. Specifically, the single-sense visual ad for healthy food results in higher advertising effectiveness than the multiple-sense visual ad. However, to draw final conclusions, further mediation analyses were necessary, as indirect mediation only can still be at hand regarding taste perceptions [48].

Table 1 shows the average number of thoughts by condition (i.e., total, positive minus negative, positive, negative, neutral, relative negative, relative positive, brand, ad and senses). The average positive, negative and neutral thoughts are calculated based on the coding of the open thought-listing task. The average total thoughts is the average of the sum of the positive, negative and neutral thoughts. The average relative negative thoughts and the average relative positive thoughts are, respectively, the average of the amount of negative or positive thoughts divided by the number of total thoughts. The results of a MANOVA with ad type as independent variable, and number of thoughts (absolute or relative) as dependent variable show that participants listed a few thoughts about the brand (*M* = 0.18, *SD* = 0.37, and *M* = 0.09, *SD* = 0.37, per participant for the single-sense and multiple-sense visual ad, respectively), but the vast majority of listed thoughts were related to the advertisement *(M* = 0.63, *SD* = 1.04, and *M* = 0.16, *SD* = 1.04, per participant for the single-sense and multiple-sense visual ad, respectively), or were sensory in nature (*M* = 0.95, *SD* = 1.018, and *M* = 1.16, *SD* = 1.02, per participant for the single-sense and multiple-sense visual ad, respectively). The mean number of total thoughts toward the healthy food visual ad did not differ between the single-sense and the multiple-sense visual ad condition (*F*(1, 80) = 1.89, *p* = 0.174). More importantly, we find that the single-sense visual ad (*M* = 0.88, *SD* = 1.80) evokes fewer negative thoughts (absolute number) than the multiple-sense visual ad (*M* = 2.14, *SD* = 1.80, *F*(1, 80) = 10.26, *p* = 0.002), whereas there is no difference between the ad types in positive thoughts (absolute number) (*F*(1, 80) = 0.95, *p* = 0.334). This result provides additional support for H2, as participants expressed fewer negative thoughts towards the single-sense visual ad for healthy food as opposed to the multiple-sense visual ad.

**Figure 4:**
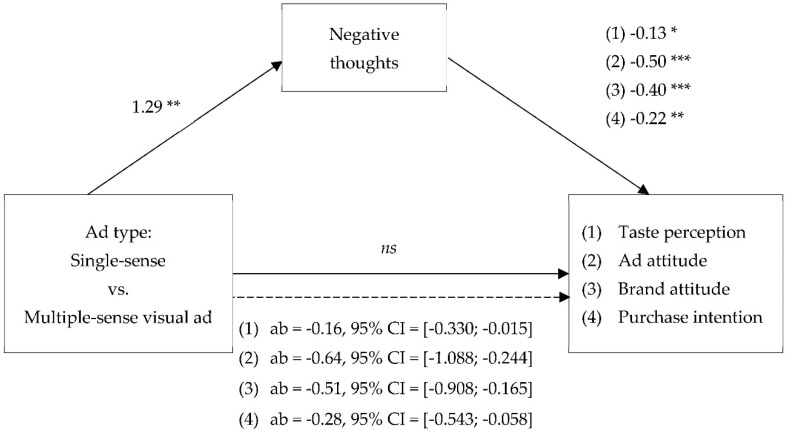
Results of Study 2. The effect of ad type on ad effectiveness is mediated by negative thoughts. Note: Other thoughts (*t*(80) >= −2.00, *p* < 0.05); *** significant at *p* < 0.001; ** significant at *p* < 0.01; * significant at *p* < 0.05.

According to the mediation analyses (model 4; Figure 4) [47], the effect of ad type on taste perceptions and ad effectiveness is mediated by the absolute amount of negative thoughts. The analyses, with 10.000 bootstraps and 95% bias-corrected intervals (CIs), reveal a significant indirect only full mediation effect for taste perceptions (ab = −0.16, *SE* = 0.73, 95% CI = [−0.330; −0.015]), and three significant full mediation analyses for Aad (ab = −0.64, *SE* = 2.02, 95% CI = [−1.088; −0.244]), Ab (ab = −0.51, *SE* = 1.74, 95% CI = [−0.908; −0.165]), and PI (ab = −0.28, *SE* = 0.1.10, 95% CI = [−0.543; −0.058]). As stated above, the effect of ad type on negative thoughts was significant (*t*(80) = 3.24, *p* = 0.002). In addition, we found significant effects of the absolute amount of negative thoughts on taste perceptions (*t*(80) = −2.21, *p* = 0.030), Aad (*t*(80) = −5.59, *p* < 0.001), Ab (*t*(80) = −5.50, *p* < 0.001), and PI (*t*(80) = −3.19, *p* = 0.002). Other thoughts as covariate was significantly related to all dependent variables (*t*(80) ≥ −2.00, *p* < 0.050). The remaining direct effects of ad type on taste perceptions (*t*(80) = −0.70, *p* = 0.485), Aad (*t*(80) = −0.55, *p* = 0.585), Ab (*t*(80) = −0.02, *p* = 0.982), and PI (*t*(80) = −0.81, *p* = 0.420) were not significant. These results provide additional support for H3. The effect of single-sense versus multiple-sense visual ads for healthy food on taste perceptions and advertising effectiveness is mediated by the negative thoughts evoked by the visual ad. Moreover, the effect of ad type on taste perceptions, Aad, Ab and PI was also mediated by the relative amount of negative thoughts. We also found a mediation effect of positive minus negative thoughts for Aad, Ab and PI. These effects, however, can be explained by the impact of negative thoughts and not by the impact of positive thoughts, as we did not find any mediation effect of positive thoughts. Next, the total amount of thoughts, relative positive thoughts and neutral thoughts did not mediate the effect of ad type on taste perceptions, Aad, Ab and PI either (Table 2). These results further support our hypothesis that in the case of healthy food, the effectiveness of ad type is mediated by negative thoughts and not by positive thoughts.

## 4. Discussion

### 4.1. Overview of the Findings

In this research, we conducted two laboratory experiments to investigate how sensory advertising can promote healthy food. Building on previous research by Elder and Krishna [17], we examined whether single-sense ads for healthy food can increase taste perceptions and advertising effectiveness compared to multiple-sense ads. We argued that this effect arises because single-sense ads for healthy food evoke fewer negative thoughts than multiple-sense ads, which is the underlying process for the positive effects of single-sense ads on taste perceptions and advertising effectiveness. 

The results of Study 1 demonstrated that for verbal sensory ads (i.e., ad slogans) displaying healthy food, taste perceptions are higher for a single-sense ad compared to a multiple-sense ad. We further showed that the healthy single-sense ad slogan evokes fewer negative thoughts compared to the healthy multiple-sense ad slogan. Moreover, these negative thoughts mediate the effect of the healthy single-sense versus multiple-sense ad slogans on both taste perceptions and advertising effectiveness. 

The results of Study 2 extended the findings from Study 1 to visual sensory ads displaying pictures of healthy food. We showed that advertising effectiveness is higher for a single-sense visual ad compared to a multiple-sense visual ad. Further, we found that the healthy single-sense ad evokes fewer negative thoughts than the healthy multiple-sense visual ad. Similar to the findings of Study 1, we find that these negative thoughts mediate the effect of the healthy single-sense versus multiple sense ad on both taste perceptions and advertising effectiveness.

### 4.2. Theoretical and Practical Contributions

To the best of our knowledge, this research is the first to investigate the effectiveness of sensory advertising for healthy food promotion. Our findings have three important contributions to the existing literature. First, we contribute to sensory perception research within food studies, by showing that referring to multiple senses in food advertising is not always more effective than referring to single senses. When promoting healthy food, single-sense ads evoke fewer negative thoughts than multiple-sense ads, and this mediates the effect of single-sense versus multiple-sense ads on taste perceptions and advertising effectiveness. In addition, we could rule out any effect of unhealthy single-sense versus multiple-sense ads on negative thoughts. These findings suggest that negative thoughts are only affected when displaying healthy food with sensory advertising, and not in the case of unhealthy food. This makes sense as we did not expect negative thoughts to be at play for unhealthy tempting foods. These results support the research by Elder and Krishna [17], as these authors showed that sensory advertisements promoting unhealthy food do not influence negative sensory thoughts, but positive sensory thoughts. As such, we reveal a different mediator to explain the effects of sensory ads promoting healthy food. 

Second, we contribute to psychological research on the effects of visuals on food consumption. Most of the information that consumers use to make food decisions is predominantly visual in nature [23,24]. Particularly, advertising elements like images, logos, pictures and videos are visual cues to communicate important features about the advertised food. We find that the effects of single-sense ads for healthy food occur when using verbal references to senses (i.e., slogans), but also in the case of visual references (i.e., ad pictures). As both verbal and visual stimuli can stimulate mental imagery [41,42,43], we show that sensory ads for healthy food can evoke sensory thoughts, either by using wording to express the senses or by using pictures to visualize them. As visual food marketing is omnipresent today and is thus gaining increasing attention, our research adds to this domain by having an immediate value for practice. Our findings confirm that the use of visual sensory references to only the taste of healthy food are beneficial for consumer attitudes toward the healthy food.

**Figure A1:**
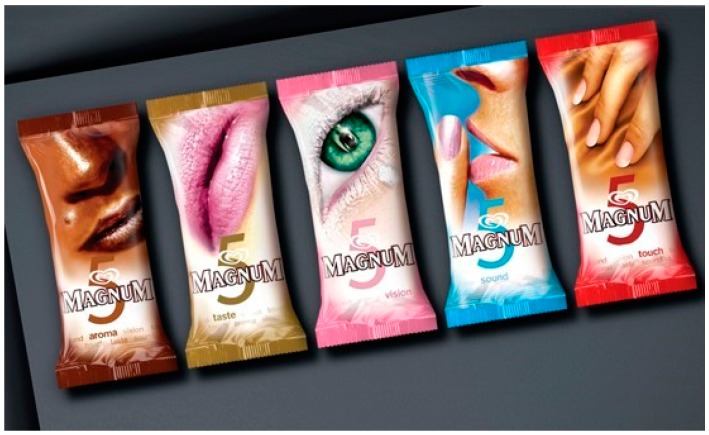
Magnum 5 Senses campaign by Unilever^©^ Magnum.

**Figure A2:**
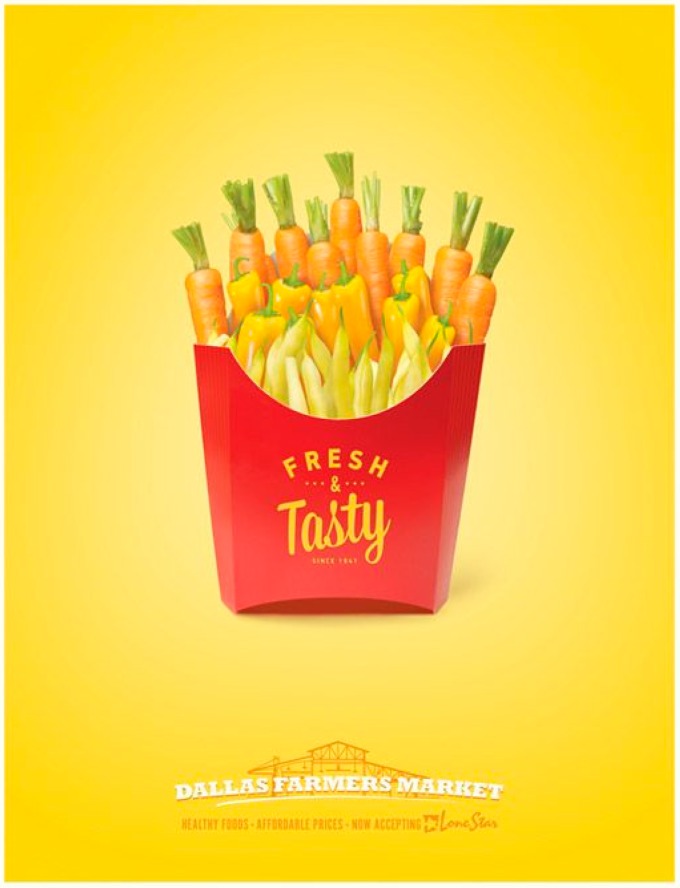
Advertisement for Dallas Farmers Market^©^ Firehouse.

Third, we add to the literature on the role of sensory marketing in driving advertising effectiveness, as we find that healthy food advertising enhances consumer attitudes toward the ad and brand, and their purchase intention, when referring only to the taste of the food, and not to multiple senses. Consequently, we provide specific practical implications for food marketers, ad executives, and public policy. Sensory advertising promoting healthy foods can create sensory triggers which appeal to the basic senses of these foods. Given today’s overload of food advertising appeals in consumers’ lives, this may be a more efficient way to engage consumers to choose healthy food. Our research thus points to the possibility of using the right kind of sensory advertising for healthy food to create favorable food product evaluations. Currently, many food manufacturers and marketers emphasize how their products appeal to the different senses, because they seem to argue that products would be better off if they were made more sensory [15,51]. For example, Unilever spent nearly 8 million dollars on an advertising campaign to promote the launch of ‘Magnum 5 Senses’, comprising Sound, Aroma, Touch, Vision and Taste variants in different flavors (Appendix A Figure A1). The ad takes you on a visual ‘journey through the senses’ to promote the consumption of Magnum ice cream. Following the findings by Elder and Krishna [17], this use of multiple-sense advertising is an effective strategy to promote unhealthy food. However, our findings indicate that referring to multiple senses in food advertising is not always more effective. Contrastingly, to promote healthy food, advertising campaigns can benefit from using sensory advertisements that mention only the taste of the healthy food instead of referring to more senses. An example of our reasoning is shown by Dallas Farmers Market, a large public market in Texas, who advertised their fresh vegetables with a visual print advertisement in which they refer to only the taste of the vegetables (Appendix A Figure A2). 

As such, our findings create relevant guidance for public policy makers who aim to stimulate healthy food consumption among consumers. Because of the increasing obesity rates among adults and children, upgrading the image of healthy food in the minds of consumers is crucial [10,11]. Most consumer food decisions are formed on the basis of experience (e.g., tastiness) and beliefs (e.g., healthiness). Policy makers have traditionally focused on healthiness, but consumption choices are primarily guided by sensory evaluations, such as taste. Our findings show that in promoting healthy food through extrinsic advertising cues, referring to taste only can actually increase taste perceptions and consumer attitudes. Applying these findings to public policy campaigns promoting healthy food can be a valuable step in the right direction to decrease obesity rates and instead increase healthy food consumption. 

While food marketing is often declared as the leading cause of the obesity epidemic [52], using successful advertising practices to promote healthy choices has the potential to enhance the health and well-being of consumers and reduce the expanding healthcare costs [30]. To summarize, our key take-away for practice is that, although multiple-sense advertising can be an effective advertising strategy, when promoting the consumption of healthy food, it is advisable to use single-sense advertising. This may stimulate a healthy shift in consumers’ food consumption and allow food manufacturers that produce healthy foods to flourish [30]. 

### 4.3. Limitations and Future Research

Further research is needed in order to further generalize the findings of the current paper. First, it is important to investigate the boundary effects of single-sense versus multiple-sense advertising in healthy and unhealthy food advertising. We have argued that intrinsic and extrinsic sensory cues associated with healthy foods, such as their taste or sight, are strong motivators of food choice, but the power of food cues on eating behavior can vary from moment to moment and from person to person. For instance, food cues are more attractive to consumers when they are hungry [53,54]. Further, cognitive processes are important determinants of our responses to food cues. An urge to consume a tempting food may be resisted if a consumer has a longer term goal of weight loss or dietary restrictions [53]. There is also evidence that responses to food cues can be facilitated or inhibited by memory processes, in the sense that processing of food information in working memory affects how much attention is paid to food cues in the environment. Similarly, memories of specific recent eating episodes play an important role in directing food choices and influencing when and how much we eat [53]. Additionally, of crucial importance in the fight against obesity is to give attention to those segments of the population who are low involved with healthy food [55]. Existing literature states that emotional advertising is particularly effective to reach low (as opposed to high) involved consumers [56,57]. We, therefore, wonder whether sensory advertising could also be effective in reaching low involved consumers. Moreover, perceived stress, depressive symptoms, social support, and family functioning can be important psychosocial factors influencing dietary intake and as such perceptions of food cues [58]. Future studies on sensory advertising for healthy food could take into account these highly relevant moderating individual difference variables.

Second, our study participants were all European. Because there are strong intercultural differences in food perception [59], it is plausible that our findings are somewhat culture-specific. Indeed, previous research shows that there are cross-cultural differences for sensory perceptions, such as taste [60]. Moreover, some studies [59,61,62] have shown that implicit and explicit associations between healthiness and tastiness are contradictory in the USA and in France. However, findings from a more recent study counter these cross-national differences in food attitudes on healthy eating [63]. These authors found that healthy food attitudes largely converged across the USA, the UK, France and Belgium. As such, it might be worthwhile to investigate whether our findings on healthy sensory advertising also apply for American or other consumers.

Third, we limited our research to one healthy food product, namely (cherry) tomatoes. In our research, we deliberately used (cherry) tomatoes as this is a healthy food but can be eaten as a snack too. In this way, we were able to compare these foods with an unhealthy food product, potato chips, which is also consumed as a snack. It remains to be tested whether our results are also generalizable for other healthy food products, including other fruits and vegetables, nuts, seeds and grains, which can be consumed as a snack or as part of a meal.

Fourth, this research focused on the single sense of taste versus multiple senses, including taste. Therefore, our studies were limited to the comparison of only taste with other senses. An important avenue for future research is to examine whether using a different sense of interest, such as smell, sound, touch, or vision, would evoke similar effects as our studies found for taste. A related area worth investigating is sensory dominance [15]. For example, could it be that taste has a greater impact on advertising effectiveness than the other senses? Or does a specific sense have a greater impact depending on the advertising medium used [16]? Similarly, future research could further delve into sensory imagery, where one sense can affect the imagery of another [15]. For instance, Lwin, Morrin, and Krishna [64] show that smell can increase visual imagery, as people had better memory of a picture if an ad with the picture also had a smell.

Fifth, in our two experimental studies, we used a verbal ad and a visual ad respectively to display sensory references. However, referring to senses can go beyond just advertising copy and can also be applied to product packaging or in-store displays [65]. For instance, the effects of using sensory labels on healthy food products remains to be explored. Some grocery retail chains have already adopted more sensory labels for their produce section. For example, using sensory labels for fruit (e.g., juicy oranges rather than Florida oranges) would inspire consumers to eat more healthy food [15]. This raises the question whether labels referring to a single-sense would similarly be more effective than labels referring to multiple-senses. Research by Van Dam and De Jonge [66] shows that negative labelling has more effect on consumer attitudes and preferences than positive labelling. It might be worth examining whether this effect holds in the context of food products. An interesting avenue for future research is, therefore, to investigate how the number of positive and negative labels per food product stimulates healthy food choices and discourages unhealthy food choices.

Sixth, the current research examined the separate effects of verbal and visual references to the senses. However, marketers can also combine wording and visuals in a single food advertisement to refer to the sensory perceptions of a food product. As most marketing and advertising communications use a combination of verbal and nonverbal information [45], future research might explore the combined effects of verbal and visual sensory references in healthy food advertising. Although we could reason that their collective effects are stronger than their individual effects, this might also potentially give rise to negative sensory thoughts due to sensory overload. Indeed, marketers using sensory advertising should not only be aware of reaching information overload, but equally of sensory overload, which can be so overwhelming that particular details of a product or experience could be missed [15]. Consequently, such sensory overload might negatively influence advertising effectiveness.

Finally, future research is required to the extent that perceived persuasion intent through sensory advertising encourages or discourages consumer behavior in the context of healthy and unhealthy food consumption. Addressing these and related research questions will further advance our understanding of how sensory advertising for healthy food impacts consumers’ thoughts, perceptions, and attitudes. 

## 5. Conclusions

The current research extends our understanding of the effects of sensory food advertising. We showed that single-sense advertisements for healthy food increase taste perceptions and advertising effectiveness compared to multiple-sense advertisements, because referring to a single sense in healthy food ads evokes fewer negative thoughts than referring to multiple senses. 

## Figures and Tables

**Table 1:** Results of Study 2. Mean number of thoughts elicited by the single-sense versus multiple-sense ad.

Type of Thoughts	Single-Sense Ad	Multiple-Sense Ad	Total	*F*-Value (1, 80)	*p*-Value
Total	4.53	5.21	4.88	1.89	0.174
Pos. minus neg.	1.10	−0.53	0.25	6.50	0.013
Positive	1.98	1.60	1.78	0.95	0.334
Negative	0.88	2.14	1.53	10.26	0.002
Neutral	1.68	1.47	1.57	0.29	0.593
Relative positive	0.41	0.31	0.36	2.06	0.155
Relative negative	0.22	0.43	0.33	7.29	0.008
Brand	0.18	0.09	0.13	0.10	0.323
Ad	0.63	0.16	1.12	17.48	<0.001
Senses	0.95	1.16	1.06	0.91	0.344

**Table 2:** Results of Study 2. Overview of mediation analyses for all types of thoughts, with 10.000 bootstraps and 95% bias-corrected intervals (CIs); Dependent variables: Taste Perception, Ad attitude (Aad), Brand attitude (Ab); Purchase Intention (PI).

Type of Thoughts	Taste Perception	Aad	Ab	PI
	ab, 95% CI	ab, 95% CI	ab, 95% CI	ab, 95% CI
Total	0.06, [−0.034; 0.212]	0.04, [−0.166; 0.269]	0.01, [−0.217; 0.206]	0.05, [−0.079; 0.215]
Pos.-neg.	−0.18, [−0.377; 0.030]	−0.56, [−1.029; −0.125]	−0.41, [−0.821; −0.079]	−0.26, [−0.529; −0.046]
Positive	−0.06, [−0.218; 0.077]	−0.15, [−0.470; 0.198]	−0.10, [−0.323; 0.133]	−0.07, [−0.258; 0.099]
Negative	−0.16, [−0.330; −0.015]	−0.64, [−1.088; −0.244]	−0.51, [−0.908; −0.165]	−0.28, [−0.543; −0.058]
Neutral	−0.02, [−0.123; 0.065]	−0.04, [−0.214; 0.131]	−0.03, [−0.189; 0.096]	−0.03, [−0.152; 0.088]
Rel. pos.	−0.09, [−0.231; 0.043]	−0.23, [−0.586; 0.104]	−0.15, [−0.340; 0.068]	−0.10, [−0.281; 0.045]
Rel. neg.	−0.22, [−0.481; −0.037]	−0.54, [−1.008; −0.143]	−0.39, [−0.755; −0.090]	0.23, [−0.493; −0.036]
